# 

**Published:** 1887-05

**Authors:** 


### Society Meetings.

### Mississippi State Dental Association.

The twelfth annual meeting of the Mississippi State Dental Association will be held on Tuesday, the 17th of May, and the two succeeding days, at Meridian, Miss.

Papers on the following subjects will be presented, viz:

Surgical Dentistry, Prosthetic Dentistry, Physiology and Histology, Pathology and Therapeutics, Materia Medica, Dental Metallurgy, Dental Chemistry, Education and Literature, Incidents of Office Practice.

On each of these subjects will follow discussions.

Clinics will be given by Dr. J. R. Knapp, of New Orleans, on Bridge Work and Gold Crowns.

Dr. J. G. McCulloch will read a paper on Microscopy, with illustrations by mounted sections.

A cordial invitation is extended to members of the profession to be present and participate in the proceedings.

Every effort is being made to make this meeting one of the most interesting and instructive ever held. The St. Elmo Hotel will entertain members at $1.50 per day during the meeting.

The State Board of Dental Examiners will hold its regular sessions during the meeting of the Association. All specially interested will please make a note of this, and be promptly in attendance.

R. J. Millin, Sect. of Board.
Jackson, Mississippi.

### Missouri State Dental Society.

The twenty-third annual meeting of the Missouri State Dental Association will be held at Kansas City, Mo., the third Tuesday in June, continuing four days—June 21st to 24th, inclusive. The programme will be of interest, and the clinical work of greater importance than ever before.

William Conrad, D. D. S.,

President.

J. G. Harper, D. D. S.,

Rec. Sec'y.

### Georgia State Dental Society.

The 19th annual meeting of this society will be held at Cumberland Island, Georgia, May 24th to the 27th, inclusive, 1887. This is one of the oldest state societies, and one of the best if we are to judge from the work it has done during all these years. The place selected for the coming meeting is a delightful one, one that of itself has great inducements for a visit to it. The island is just off the coast of the southeast corner of Georgia. There will be found all the tropical beauties and richness.

The programme for the occasion is a very inviting one; papers will be read upon Physiology and Etiology, Histology and Microscopy, Pathology and Surgery, Chemistry and Therapeutics, Operative Dentistry, Prosthetic Dentistry, Dental Education and Literature. Several voluntary papers and essays will also be presented. The names attached to these various subjects upon the list is sufficient evidence of the character of the papers, and the value of the matter they will contain. In addition to these regular subjects, there will be special topics, namely: Pathology of Caries, by E. Parsons; The Different Filling Materials, G. H. Winkler; Anæsthesia in Dentistry, B. H. Catching; Alveolar Abscess, W. F. Tignor; Cohesive Gold, T. M. Allen; New Processes, S. A. White; Nerve Canals, L. D. Carpenter; Gold and Porcelain Crowns, A. G. Bouton; Electric Mallet, Wm. I. Crenshaw; The Merits of Bridge Work, J. H. Coyle; Capping Nerves, J. H. Moncrief; Artificial Dentures, the best, J. S. Thompson; Incidents of Office Practice, J. M. Mason.

The Committee of Arrangements have secured reduced railroad fare throughout the State of Georgia, which will be full fare going, and one-third fare returning. Hotel rates at the Island, $1.50 per day. An ocean excursion is also provided for. The effort of the Committee has been untiring to make it a pleasant and prof-

Every city having ten or more dentists should have a good and successful local society, and good comfortable quarters should be one of the first things to receive attention. Long may the Detroit Dental Society live and prosper.

### Georgia State Dental Society.

itable occasion. It is to be hoped that every member of the profession in the State of Georgia, so far as practicable, will be present, and a cordial invitation is extended to the profession of neighboring states.

The Board of Dental Examiners will meet at the Ocean Hotel, in Brunswick, on Monday, May 23rd, at 10 o'clock, A. M., for the examination of all candidates who may desire to be authorized to practice, under the law of the State. The law is very explicit, as is indicated by the following paragraph: "Any person commencing the practice of Dentistry, from and after the passage of this act, (approved October 9th, 1885,) shall be deemed guilty of a misdemeanor, unless such person shall have obtained license from the Board of Examiners, duly authorized to grant said license." All persons interested are expected to appear promptly before the board at its first session.

L. D. CARPENTER,
SECRETARY.

### The Kentucky State Dental Association.

The Kentucky State Dental Association will hold its 17th annual meeting in the Louisville College of Dentistry, Louisville, Kentucky, beginning June 7th, and continuing three days.

The following subjects have been assigned to discussion, namely: 1. "The Proper Care of Children's Teeth." 2. "The Consistent Saving of Time in Prolonged Operations—How can it best be accomplished?" 3. "The Cause of Dental Caries, and the Best Preventive Treatment." 4. "The Proper Treatment of Pulpless Teeth." 5. "Crown and Bridge Work." 6. "New Remedies." 7. "Incidents of Office Practice." Papers have also been promised upon "Scirrhus Gangrene of the Inferior Maxillary," "First Dentition," and "Diagnosis of Early Diseases, Irregularities," "The Rationale of Crown and So-Called Bridge Work," "Inflammation," and in addition to these, a good series of clinics will be given, embracing the general features of every day practice. Arrangements have been made for one of the best meetings this body has ever held. Reduced rates have been secured from the best hotels of Louisville, ranging from $1.50 to $2.50 per day. It is also hoped that some reduction in railroad fare may be secured. There will be a large display of new and interesting instruments and appliances.

### State Board of Dental Examiners.

This board will meet daily, during the session of the Association, to examine applicants and register names of Dentists not already registered. The precise hour and place of holding these sessions of the Board, will be determined at the time. All interested, however, should be promptly on hand.

### Southern Dental Association.

The annual meeting of this body will be held at Old Point Comfort, Virginia, on Tuesday, the 30th of August, to the 2nd of September, inclusive.

A most excellent programme has been prepared, which embraces the following subjects, namely: Dental Education, Hygiene, Pathology and Therapeutics, Histology and Microscopy, Chemistry, Operative Dentistry, Mechanical Dentistry, Literature, and voluntary essays. Men of acknowledged ability have been placed in charge of these different subjects, and interesting and instructive papers will be read upon each. A large number of clinical operators have been secured, who will present a great variety of methods and processes, in matters embracing not only the methods of operation, but the instruments and appliances employed. Many of the most noted clinicians in the country have been secured for the occasion, and all who attend, may be well assured of having ample opportunity for observation, and gaining new points and principles in practice.

The Executive Committee, with Dr. J. Hall Moore, of Richmond for its Chairman, has spared no pains and labor to secure the most perfect system of clinical demonstrations that has ever been held.

The place for the meeting is one especially inviting. It is a very popular resort, and justly so because of its many attractions.

A week spent there just previous to the meeting of the International Medical Congress will tone up and prepare for the duties of the Congress the succeeding week, in Washington. With the oldest living graduate of Dental Surgery, Dr. W. W. H. Thackston, for its President, and Dr. J. Hall Moore, Chairman of the Executive Committee, and the strong and efficient men upon each of the other committees, this meeting must be a grand one, which beyond doubt will secure greater results than have ever accrued from the meeting of this body hitherto.

Then let all go who can, prepared to contribute a little, and receive much, for there will be much to be given, without money and without price.

### Illinois State Dental Society.

THE twenty-third annual meeting of the Illinois State Dental Society will be held at Jacksonville, Ill., beginning Tuesday, May 10th and continuing four days.

A full programme of scientific work is arranged for, including a large and instructive clinic. Dr. Black will give short lectures on micro-organisms, with practical demonstrations of their culture. All dentists will be cordially welcomed.

J. W. WASSALL, Secretary.

### Northern Ohio Dental Association.

The twenty-eighth annual meeting will be held in Cleveland, Ohio, Tuesday and Wednesday, May 10th and 11th, 1887, commencing at 10 a. m. Tuesday. Meeting to be held at Cogswell's Dental Depot, No. 29 Euclid avenue. A cordial invitation is extended to all the profession, J. R. Bell, President; S. B. Dewey, Corresponding Secretary.

Order of Business.—1st. Reading the Minutes. 2d. Reports of Officers and Committees. 3d. Reading of Essays. 4th. Discussions. Electiou of officers Wednesday 10 a. m.

Subjects for Discussion.—1. The Relative Duty of Patient and Operator. Paper by Dr. J. W. Lyder, of Akron. 2. Best Treatment for Bucal and Labial Cavities. Paper by Dr. J. E. Robinson, of Cleveland. 3d. Artificial Substitutes for Natural Teeth. Paper by Dr. H. Barnes, of Cleveland.

### Wisconsin Dental Society.

Wisconsin State Dental Society meets annually. Next meeting at Milwaukee, July 19th, 1887. President, W. H. Chelson, Appleton; Secretary, W. S. Sullivan, Madison, Wis.

### THE DENTAL REGISTER,

**A MONTHLY MAGAZINE DEVOTED TO THE INTERESTS OF THE**

### DENTAL PROFESSION.

***Terms Reduced to* $2.00 *Per Year. Single Copies,* 25 *Cents.***

### EDITED BY PROF. J. TAFT, D.D.S.

—AND—

### Published by SAM'L A. CROCKER & CO., Ohio Dental and Surgical Depot.

The volume commences in January and closes in December.

The REGISTER being issued monthly is always fresh, therefore Indispensable to the practitioner who desires to keep posted up with the new inventions and improvements which are daily developed. Neither expense nor effort will be spared to give value and variety to its contents. Articles will be illustrated with well executed engravings whenever they will add to the interest or assist to explanation.

Its extensive circulation and frequency of issue make it one of the BEST DENTAL ADVERTISING MEDIUMS in the United States.

All subscriptions must begin with January or July.

Local or special notices, five lines or less, will be inserted for $3 each insertion; over five lines, 50 cts. per line.

All advertising bills payable in advance, or at furthest, after the issue of the first number containing the advertisement. All transient advertisements to be paid for in advance.

☞CONTRIBUTIONS SOLICITED.

Exclusively Editorial Communications should be addressed to the Editor.

The latest number of the REGISTER may be ordered with or without other goods at any time, and all letters should be addressed to

**SAM'L A. CROCKER & CO., Ohio Dental and Surgical Depot, Cincinnati, O.**

